# Kinematics and muscle activity of pectoral fins in rainbow trout (*Oncorhynchus mykiss*) station holding in turbulent flow

**DOI:** 10.1242/jeb.246275

**Published:** 2024-03-12

**Authors:** Brendan J. Gibbs, Otar Akanyeti, James C. Liao

**Affiliations:** ^1^ Department of Biology, The Whitney Laboratory for Marine Bioscience, University of Florida, St Augustine, FL 32080, USA; ^2^ Department of Computer Science, Aberystwyth University, Aberystwyth, Ceredigion SY23 3DB, UK

**Keywords:** Fish, Electromyography, Fluid dynamics, High-speed video, Control surfaces, Locomotion

## Abstract

Pectoral fins play a crucial role in fish locomotion. Despite fishes living in complex fluid environments that exist in rivers and tidal flows, the role of the pectoral fins in navigating turbulent flows is not well understood. This study investigated the kinematics and muscle activity of pectoral fins in rainbow trout as they held station in the unsteady flows behind a D-section cylinder. We observed two distinct pectoral fin behaviors, one during braking and the other during Kármán gaiting. These behaviors were correlated to whole-body movements in response to the hydrodynamic conditions of specific regions in the cylinder wake. Sustained fin extensions during braking, where the fin was held out to maintain its position away from the body and against the flow, were associated with the cessation of forward body velocity, where the fish avoided the suction region directly downstream of the cylinder. Transient fin extensions and retractions during Kármán gaiting controlled body movements in the cross-stream direction. These two fin behaviors had different patterns of muscle activity. All braking events required recruitment from both the abductor and adductor musculature to actively extend a pectoral fin. In contrast, over 50% of fin extension movements during Kármán gaiting proceed in the absence of muscle activity. We reveal that in unsteady fluid environments, pectoral fin movements are the result of a complex combination of passive and active mechanisms that deviate substantially from canonical labriform locomotion, the implications of which await further work on the integration of sensory and motor systems.

## Teaser

**Summary:** Synchronized high-speed video and electromyography of pectoral fins in station-holding trout reveal active and passive control.

## INTRODUCTION

Animals use their contracting muscles as motors, exerting a force on the external environment to enable locomotion (Cavagna et al., 1977; Biewener, 1989; Winters et al., 2000; Dickinson et al., 2000). However, muscles can exhibit a wide range of functions even when moving across relatively uniform environments, acting as brakes, springs and struts (Cavagna et al., 1977; Ker et al., 1987; Biewener and Blickhan, 1988; McMahon and Cheng, 1990; Dickinson et al., 2000; Winters et al., 2000; Daley et al., 2007). For example, the wing control muscles of the fly Diptera generate little to no power but instead act as springs to direct the mechanical energy from larger power muscles into complex wing motion (Tu and Dickinson, 1994; Dickinson and Tu, 1997). In cockroaches (*Blaberus discoidalis*), leg muscles are capable of power production but also act as brakes during running to slow leg swing (Full et al., 1998). The hindlimb muscles in hopping wallabies (*Macropus eugenii*) can contract isometrically during locomotion, serving as struts to transfer forces to their elastic tendons to enable efficient energy storage and release (Biewener et al., 1998). External properties of the environment, such as uneven terrain or unsteady flows, can also interact with moving animals (Liao et al., 2003b; Fish and Lauder, 2006; Biewener and Daley, 2007; Taguchi and Liao, 2011). The variability of environmental substrates profoundly affects the control during organismal movement (Ferris et al., 1998; Lejeune et al., 1998; Dickinson et al., 2000; Biewener, 2003; Daley et al., 2007; Li et al., 2013). This further complicates the ability to understand locomotory mechanisms of animals in a natural context. For example, during land running, the legs of basilisk lizards (*Basiliscus plumifrons*) act as springs to both produce force and store elastic energy (Hsieh, 2003), but on water their legs transition to act more like pistons (Hsieh and Lauder, 2004). Swimming fishes can interact with unsteady flows to drastically change the energetic requirements of locomotion. However, the function of the fin muscles underlying these changes in performance remain unresolved (Liao et al., 2003b; Beal et al., 2006; Taguchi and Liao, 2011).

Predominant insights into locomotion often focus on movement in uniform environments, as the variability inherent in muscle function makes it challenging to understand how animals move in complex conditions. Yet locomotion has evolved in complex physical environments, which characterize the natural habitats where animals have evolved to perform. This is especially the case for animals that live in aquatic habitats where the surrounding fluid provides a three-dimensional substrate. Fluids can exert complex forces that influence the powered and passive movements of animals needed to dynamically control locomotion (Fish and Lauder, 2006). Fish use their fins and body as control surfaces both to maintain stability and to execute maneuvers (Geerlink, 1986; Wilga and Lauder, 2000; Lauder and Drucker, 2002, 2004; Webb and Weihs, 1994). One of the most prominent control surfaces in fish are the paired pectoral fins, which are flapped in a mechanism similar to the wing beat cycle of birds during labriform locomotion (Gibb et al., 1994; Walker and Westneat, 1997, 2002; Drucker and Lauder, 2000). For example, surfperches (Embiotocidae) oscillate their pectoral fins to generate positive and negative lift forces (Webb, 1973; Drucker and Jensen, 1996).

Studies revealing simultaneous fin kinematics and muscle activity have laid the foundation to understand how the abductor and adductor muscles are used to power pectoral fin motion (Westneat et al., 2004). However, these studies typically investigate labriform swimmers in relatively uniform hydrodynamic conditions (Westneat, 1996; Drucker et al., 2005). While almost all fish species use their pectoral fins in some manner for propulsion and maneuverability, the majority are not labriform swimmers. Kinematic studies of non-labriform swimmers in uniform flows have shown that pectoral fins are crucial in initiating maneuvers and maintaining dynamic stability (Geerlink, 1986; Arnold et al., 1991; Wilga and Lauder, 2000; Webb, 2002; Drucker and Lauder, 2003), but their activation in complex flows is far less understood.

In nature, rainbow trout must feed, migrate and spawn in the turbulent flows of rivers and streams. We investigated the role of their pectoral fins during station holding by experimentally generating a von Kármán vortex street behind a D-section cylinder. In flows, the movements and postures of the pectoral fins can add a dimension to the control of locomotion that does not otherwise exist for fish swimming through still water (Webb, 1984, 2002). Here, we tested the hypothesis that pectoral fin muscle activity is strongly correlated to fin movements when trout swim in a von Kármán vortex street. Our results suggest that fin movement in turbulent flows can occur with or without muscle activity, deviating from paired fin studies in steady flow (Westneat, 1996; Rosenberger and Westneat, 1999; Westneat et al., 2004; Standen, 2010).

## MATERIALS AND METHODS

### Animals

Adult rainbow trout, *Oncorhynchus mykiss* (Walbaum), were obtained from a commercial hatchery (Wolf Creek National Fish Hatchery, Jamestown, KY, USA). Fish were held in a 500 l freshwater tank (maintained at 15±1°C) with a constant flow and were fed commercial trout pellets daily. All experimental procedures were approved by the University of Florida Institutional Animal Care and Use Committee (IACUC) (ID 202200000056).

### Electrode construction

Polyimide insulated stainless steel (0.005 cm diameter; California Fine Wire Co., Grover Beach, CA, USA) was used to construct electrodes to measure muscle activity. Electrodes were constructed by threading one of the stainless steel wires through the barrel of a hypodermic needle (26 gauge 5/8) and the tips of the wires were then stripped and rolled into a hook with micro-forceps (Liao, 2004).

### Surgery

**Fig. 1.**
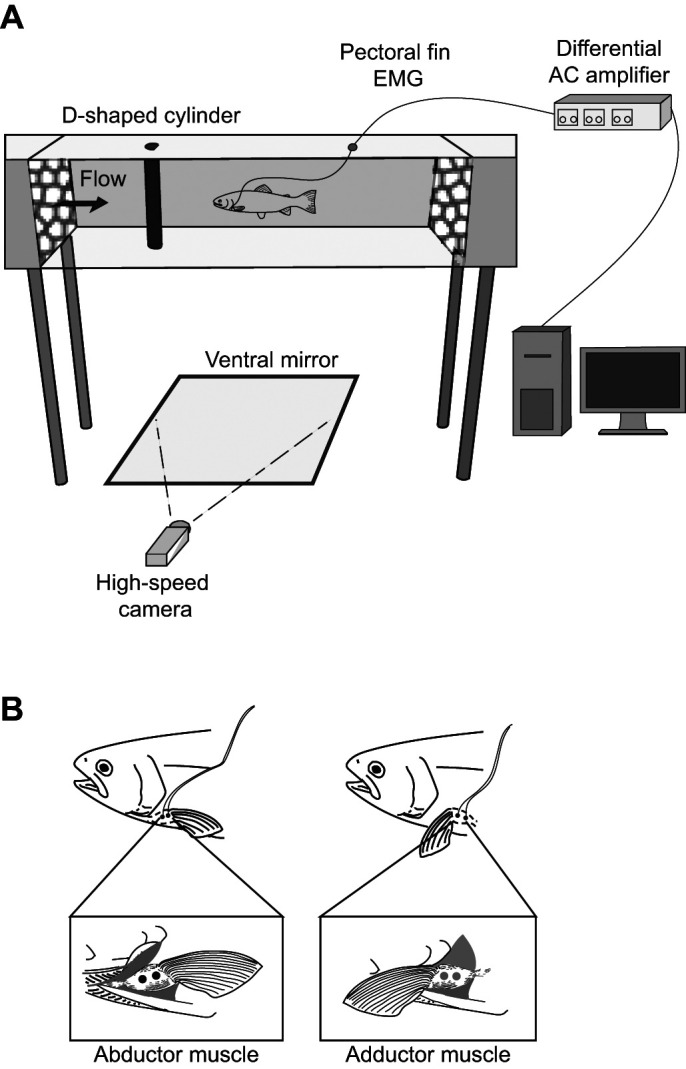
**Experimental overview.** Rainbow trout were placed in a variable speed flow tank to simultaneously record (A) swimming kinematics and (B) pectoral fin muscle activity.

Rainbow trout were anesthetized in a 2.6 l tank (15±1°C) containing a solution of 0.042 g l^−1^ tricaine methanesulfonate (MS-222; Finquel Inc., Argent Chemical Laboratories Inc., Redmond, WA, USA) which was buffered with sodium bicarbonate. Once fish were unresponsive, they were transferred to a 9.25 l surgery tray where fresh oxygenated water containing a dilute amount of MS-222 (0.03 g l^−1^ of MS-222 at 15±1°C) was recirculated over the gills. For both left and right pectoral fins, two electrodes were inserted subdermally (∼2 mm deep) into the mid-belly of the abductor and adductor musculature (Fig. 1B). After implantation on both fins, all eight wires were glued into one cable with cyanoacrylate and anchored with a suture loop (4-0 gauge braided silk thread; Ethicon Inc., Somerville, NJ, USA) anterior to the dorsal fin to prevent electrodes from being pulled out during the experiment. After surgery (∼20 min), fish were transferred to a 10 l freshwater tank to recover until a righting response was observed. Fish were then placed in the flow tank (850 l at 15±1°C) and allowed to recover for 1 h before the experiment started. The electrodes were dissected out of the abductor and adductor muscles after the experiment to confirm their placement.

### Experimental procedures

Experiments were performed on trout [*n*=5 fish, mean±s.e.m. body length (*L*) 21.7±0.837 cm] swimming behind a stationary, 5 cm diameter, polyvinyl chloride D-section cylinder. Trout held station in the von Kármán vortex street (Fig. 1A) behind the cylinder at five experimental flow speeds (45–85 cm s^−1^; 2–3.75 *L* s^−1^), corresponding to a range of Reynolds numbers (*Re* 2.5×10^4^ to 4.8×10^4^), vortex shedding frequencies (∼2–5 Hz) and vorticity values (∼1×10^−4^ to 7×10^−4^ m^2^ s^−3^) (Stewart et al., 2016) (Fig. S1).

Electromyography (EMG) signals were recorded with a Powerlab 16/35 analog to digital converter (AD Instruments Ltd, Dunedin, New Zealand) at a sampling rate of 1000 kilosamples s^−1^. Signals were amplified (10 k gain) using a model 1700 differential AC amplifier (AM Systems, Sequim, WA, USA) with a 60 Hz notch filter, a 100 Hz low cutoff and a 3000 Hz high cutoff (Jayne and Lauder, 1995; Liao, 2004). Simultaneous high-speed video was taken with EMG recordings to quantify swimming kinematics of the fin and the body. A Phantom Miro LAB 340 high-speed camera (150 frames s^−1^; Vision Research, Wayne, NJ, USA) and a front surface mirror below the working section of the flow tank allowed for ventral recordings of the trout as it swam behind the cylinder (Fig. 1A).

### Kinematic analysis

Whole-body and pectoral fin movements were digitized using DeepLabCut (Mathis et al., 2018), which allows for 3D markerless pose estimation through the training of a deep neural network. Five points on each pectoral fin [two at the fin base (points 1 and 5), one at the distal-most point of the leading edge (point 2), one at the distal-most point of the trailing edge (point 4), and one point at the longest radial (point 3)] were used to quantify fin movement as well as seven points on the midline (snout, gill arch, pectoral fin midpoint, pelvic fin girdle, anal fin, caudal fin base, caudal fin tip) to derive whole-body kinematics (Fig. S2). One-hundred frames from behavioral sequences of interest were manually digitized to train a neural network through 800,000 iterations. Analyzed videos were exported to MATLAB where custom scripts plotted pectoral fin extension against body velocity.

All digitized midline and pectoral fin points were visually inspected to ensure data quality. Datasets with outliers (where DeepLabCut predictions were not accurate) were manually corrected. To reduce digitization noise, these points were further smoothed using a moving average filter with a window size set to 20% of frame rate, which was determined through trial and error after analyzing several datasets. We chose this filtering method rather than other smoothing approaches used in fish kinematics analysis (e.g. quintic spline interpolation and Butterworth filter) so that our results were less sensitive to unexpected jumps or frame-by-frame fluctuations in the data. One drawback of this approach is that it provides a conservative estimation of the maximum and minimum points.

To quantify pectoral fin kinematics, we measured fin extension as the perpendicular distance from the tip of the fin (third pectoral fin point) to the base (the line at the intersection between the first and fifth pectoral fin points) with maximum distance corresponding to the fully extended fin. We assumed that an extension event occurred if the distance was greater than or equal to 0.05 *L*, and the duration of the event was estimated as the time difference between the onset and offset time points.

Through the analysis of fish midline kinematics, the forward and lateral velocity of the body was estimated by tracking the third midline point, which was close to the center of mass (COM). We also divided swimming sequences into two behavior groups: Kármán gaiting and braking. Kármán gaiting video sequences were identified using five criteria described in Akanyeti and Liao (2013). Briefly, these criteria were: (1) the fish was holding station and did not drift upstream or downstream, (2) there was a traveling wave along the body, (3) the body displayed a large lateral displacement (>0.5 *L*), (4) the body adopted a long wavelength (>1 *L*) and (5) there were no transient small-amplitude, high-frequency tail beats. Braking video sequences were identified using the following criteria: (1) the fish was drifting upstream, (2) the fish had a forward acceleration followed by an immediate deceleration, (3) the fish did not exhibit steady swimming kinematics and (4) the fish did not exhibit whole-body undulation observed during forward acceleration (Akanyeti et al., 2017).

We first investigated where (relative to the cylinder) and at what flow speeds fish exhibited these two behaviors. To study whether fin activity differed between the two behaviors, we measured the synchronicity between left and right fin events, assuming that the two fins were simultaneously extended if they had 50% overlap based on their onset and offset points. We hypothesized that the fish use the fins to decelerate during braking. To begin to investigate the potential benefits of fin use for each behavior (for instance, increasing hydrodynamic drag to decelerate faster while braking), we studied changes in body velocity (both forward and lateral) before and after the fin extension event. For each event, we compared the pre- and post-velocity, averaged over 0.2 s windows separated by the time point at which the fin was fully extended. To confirm that the change in pre- and post-velocity was statistically significant, we first checked for normality using a one sample Kolmogorov–Smirnov test and then performed a Student's *t*-test.

### EMG analysis

Muscle activity during experimentation was captured through the software LabChart (AD Instruments Ltd). EMG signals from corresponding kinematic behavioral sequences were initially analyzed with a 10th order Butterworth filter (Liao, 2004) to filter out noise. An in-house MATLAB script used a threshold analysis to determine relevant muscle signals. EMG signals that were at least 25% of the mean spike amplitude for a given electrode were determined to be relevant muscle signals. Raw traces were also manually inspected to ensure that spikes were not artifacts. As a control, EMG activity was recorded in trout swimming in steady flow before the cylinder was introduced. During this time, the pectoral fins were adducted against the body (Drucker and Lauder, 2003) and no EMG activity was observed.

## RESULTS

### Behavior in a von Kármán vortex street

**Fig. 2.**
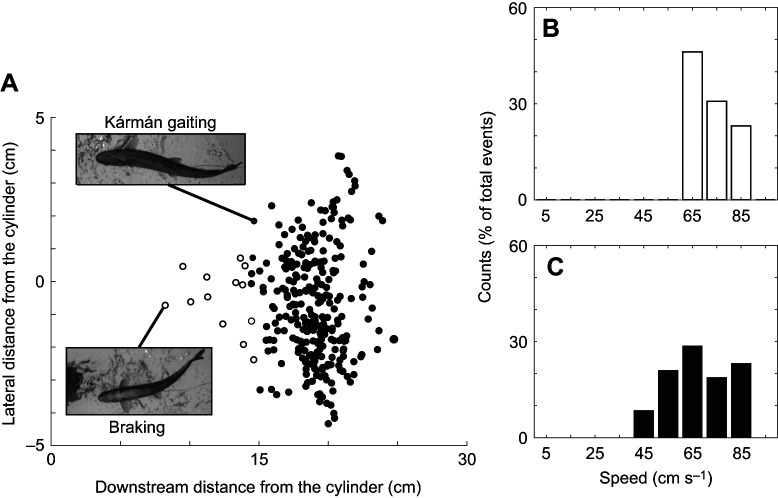
**Behavior in a von Kármán vortex street.** (A) Spatial distribution of our two observed behaviors, braking and Kármán gaiting, relative to the 5 cm D-shaped cylinder (0,0). (B,C) Percentage of braking and Kármán gaiting events across five different flow speeds. (B) Braking events were observed at only the highest three speeds (65–85 cm s^−1^) while (C) Kármán gaiting was observed at all tested flow speeds (45–85 cm s^−1^).

Rainbow trout used their pectoral fins during two behaviors, braking and Kármán gaiting, when positioned around the von Kármán vortex street. We observed that braking behaviors generally consisted of the abduction of the pectoral fins along the fish's long axis (Drucker and Lauder, 2003) as the fish were pulled into the D-shaped cylinder, while Kármán gaiting behaviors consisted of any pectoral fin extensions that occurred during Kármán gait swimming bouts (Liao et al., 2003a). These two behaviors occurred at two different locations in the vortex street (Fig. 2A). Kármán gaiting generally occurred further downstream than braking behavior, with the majority of events occurring at 17–20 cm (0.78–0.92 *L*) downstream of the cylinder. Many more Kármán gaiting events were observed (*n*=272) than braking events (*n*=13), with Kármán gaiting having a much greater lateral position across the entire von Kármán vortex street (−4.5 to 4 cm) (Fig. 2A). Kármán gaiting bouts were observed at all tested flow speeds (45–85 cm s^−1^; 2.1–3.9 *L* s^−1^) (Fig. 2C).

Braking behavior was only observed at higher flow speeds (65–85 cm s^−1^; 3–3.9 *L* s^−1^) (Fig. 2B). Braking events occurred approximately 7–15 cm (0.32–0.69 *L*) downstream of the D-shaped cylinder and within 3 cm (0.14 *L*) laterally of the cylinder. All braking behaviors occurred as the trout entered the area of low pressure directly behind the D-shaped cylinder. This area of low pressure creates a suction zone which attracts the body of a fish whenever it is in close proximity (Zdravkovich, 1997; Liao et al., 2003a; Beal et al., 2006).

### Synchronized left and right side pectoral fin kinematics

**Fig. 3.**
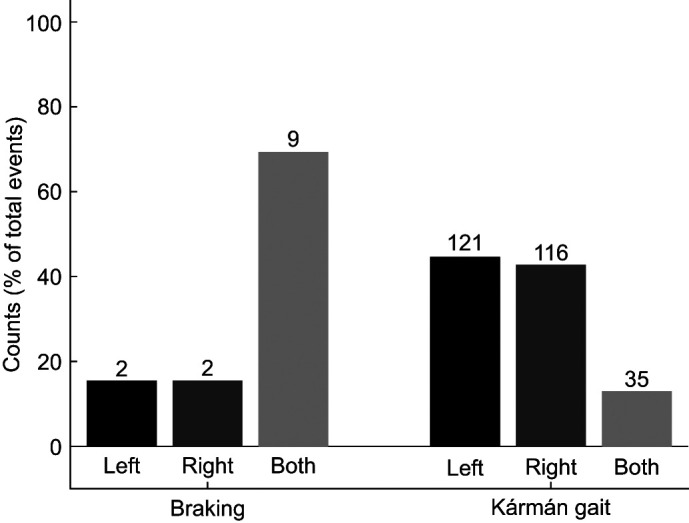
**Relative counts of synchronous and asynchronous pectoral fin use for braking and Kármán gaiting behaviors.** Pectoral fin use during braking was synchronized over 60% of the time (9 of 13 total events) while pectoral fin use during Kármán gaiting was asynchronous over 80% of the time (237 of 272 total events).

**Fig. 4.**
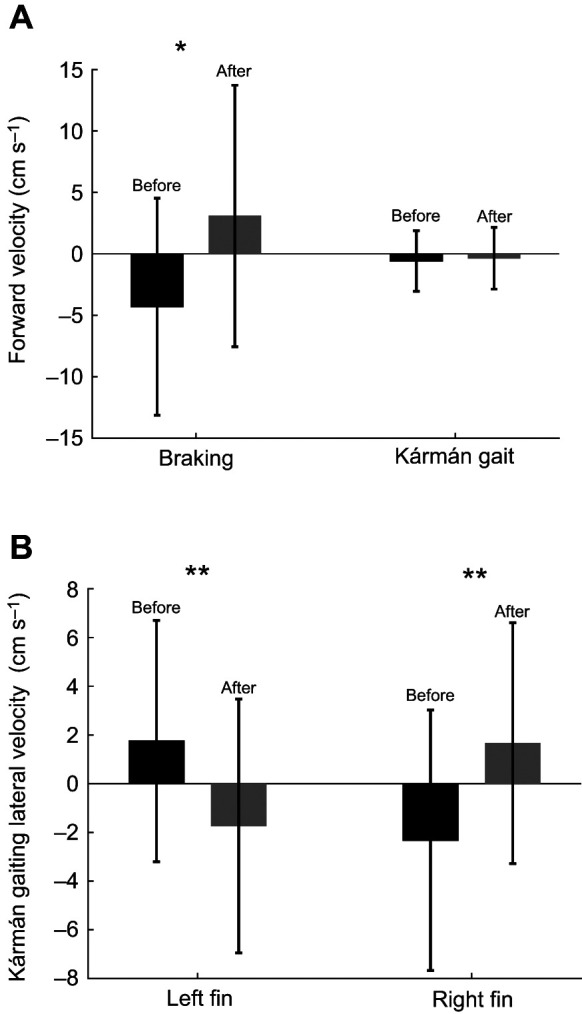
**Whole-body forward and lateral velocity changes after a fin extension.** (A) Forward velocity of the trout's center of mass (COM) prior to and after a braking event. During braking behaviors, forward velocity went from negative to positive (*t*-test, *P=0.02) after fin extension. Fin extension during Kármán gaiting had no significant effect on whole-body forward velocity (*t*-test, *P*>0.05). (B) Lateral velocity of the COM of a trout before and after an extension of a single pectoral fin while Kármán gaiting. The extension of a fin during Kármán gaiting shifts the whole-body lateral velocity towards the extended fin (**P<0.001).

We observed that pectoral fin kinematics varied between behaviors. The most apparent kinematic difference between our two observed behaviors was the synchronicity of fin abduction. During Kármán gaiting bouts, the pectoral fins were used asynchronously over 80% of the time (237 of 272 total events) (Fig. 3). These extensions had no significant effect on the fish's whole-body forward velocity [prior to fin extension −0.6±0.04 cm s^−1^ (0.03±0.002 *L*); post-fin extension −0.4±0.04 cm s^−1^ (0.02±0.002 *L*); *t*-test, *P*=0.17] (Fig. 4A). Conversely, pectoral fin extensions during Kármán gaiting had a significant effect on the lateral velocity of the fish (Fig. 4B). Prior to the extension of the left pectoral fin, the trout's lateral velocity was a mean (± s.e.m.) of 1.8 ± 0.30 cm s^−1^ (0.08 *L* s^−1^). After the left fin was fully extended, whole-body lateral velocity shifted to −2.3 ± 0.32 cm s^−1^ (0.11 *L* s^−1^). The same trend was seen during right fin extensions as prior to fin abduction, the whole-body lateral velocity was −1.7 ± 0.31 cm s^−1^ (0.08 *L* s^−1^) but after full extension, lateral velocity increased to 1.7 ± 0.29 cm s^−1^ (0.08 *L* s^−1^). These results illustrate that a single pectoral fin extension during Kármán gaiting may facilitate lateral movement towards the abducted fin.

During braking, both fins were simultaneously abducted over 60% of the time (9 out of 13 total events) (Fig. 3). This extension of the pectoral fins allowed the trout to effectively stop in place. Prior to fin extension, the trout's COM velocity was −4.3±2.4 cm s^−1^ (0.20 *L* s^−1^), indicating forward motion towards the cylinder. After extension of both pectoral fins, the trout's COM velocity shifted to 3.1±0.16 cm s^−1^ (0.14 *L* s^−1^). Extension of the pectoral fins during braking allows trout to stop their forward momentum to drop back in the flow away from the cylinder (Fig. 4A).

### Muscle activity during braking

**Fig. 5.**
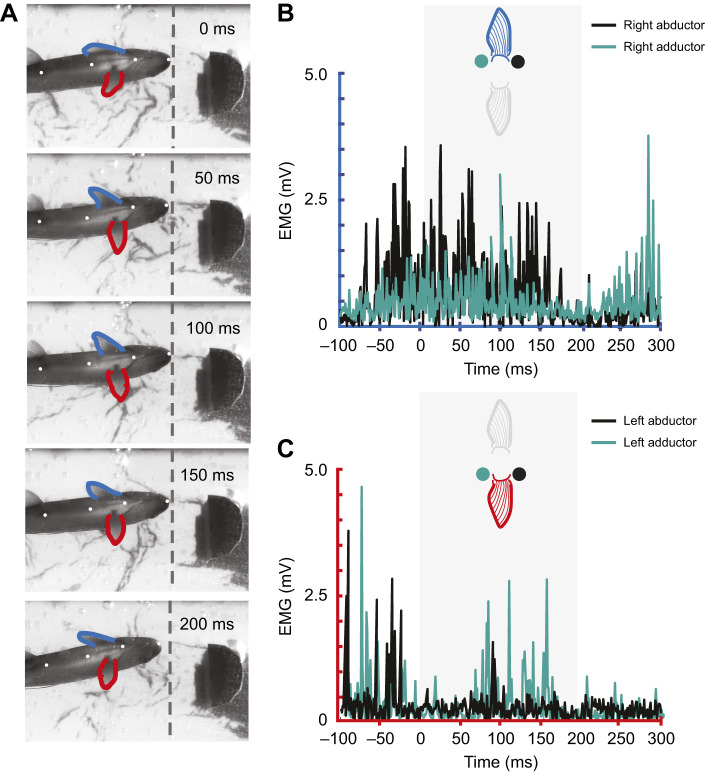
**Visual kinematics and corresponding electromyography recordings from the abductor and adductor musculature of both fins during a braking event.** (A) Ventral sequence of braking shown in 50 ms intervals, where red indicates the fish's left pectoral fin and blue indicates the right pectoral fin; 100 ms is the point of maximal pectoral fin extension and the dashed line at the fish's snout represents the trout's forwardmost point relative to the cylinder. At the subsequent 50 ms intervals after maximal pectoral fin extension, the trout begins to drift downstream of its forwardmost point. (B,C) Electromyography (EMG) traces from the abductor (black) and adductor musculature (teal) during the braking sequence observed in A. Prior to the first frame (0 ms), activity is observed from both fins’ abductor musculature, which is likely responsible for forward fin extensions. Adductor activity from both fins is highest as the fins are held out against the flow (100 ms). Differences in patterns of muscle activation between the two fins (B,C) are likely due to the asymmetrical approach of the fish relative to the cylinder (A).

Braking behaviors were facilitated through active muscle recruitment (Table 1, onset). An example of a stereotypical braking sequence is shown in Fig. 5A with corresponding EMG traces in Fig. 5B,C. In the EMG trace of the left pectoral fin (Fig. 5C), the abductor musculature shows activity at approximately −90 ms and −40 ms, which is prior to the behavioral sequence illustrated by Fig. 5A. These spikes represent the initial recruitment of the abductor musculature and are responsible for the abduction of the left pectoral fin prior to the first frame (0 ms). As the fish approached the cylinder, the left abductor once again was active (spike at 90 ms) and this activity was responsible for the full extension of the left fin. Activity from the adductor musculature was also observed throughout braking sequences. In Fig. 5C, adductor activity was highest at 60–160 ms, which is the time frame that the trout's left pectoral fin was fully extended and fighting against the flow. The right pectoral fin (Fig. 5B) followed the same pattern observed in the left fin as abductor activity is correlated with fin extensions and adductor activity corresponds to holding the fin steady against the flow.

**Table 1.**
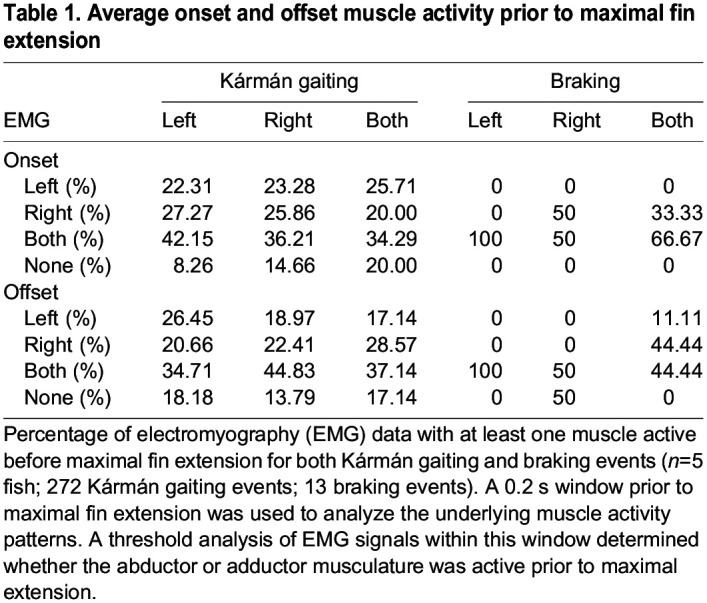
Average onset and offset muscle activity prior to maximal fin extension

### Muscle activity during Kármán gaiting

**Fig. 6.**
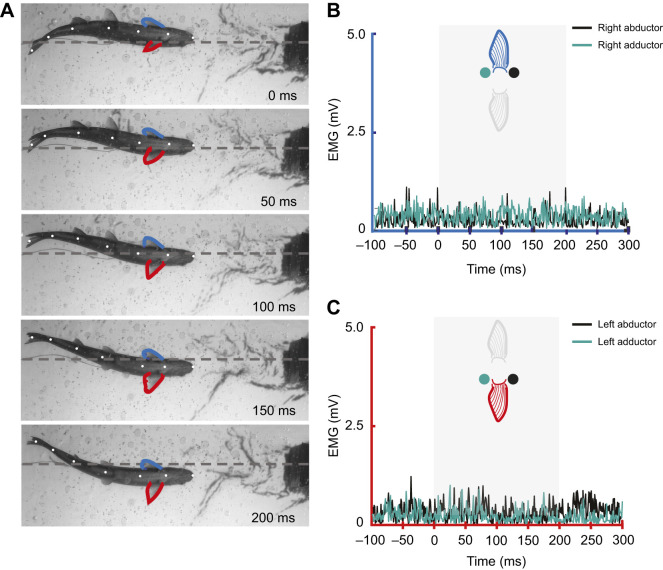
**Visual kinematics and corresponding EMG recordings from the abductor and adductor musculature during a Kármán gaiting event.** (A) Ventral sequence of Kármán gaiting shown in 50 ms intervals where red indicates the fish's left pectoral fin and blue indicates the right fin. The horizontal dashed line is the centerline of the cylinder and it can be observed that the left pectoral fin (red) is abducted away from the body during this Kármán gaiting sequence. (B) EMG traces from the left and right pectoral fin musculature. No muscle activity was observed in the left or right pectoral fin during this sequence.

**Fig. 7.**
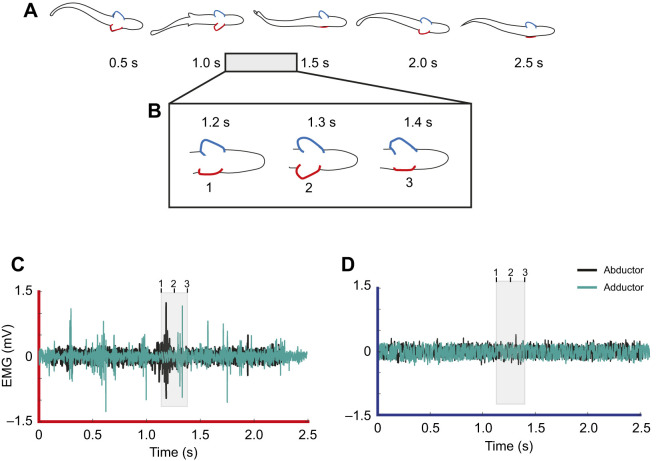
**Pectoral fin and whole-body kinematics with corresponding left and right pectoral fin muscle activity for a trout Kármán gaiting over a 2 s interval at a flow speed of 65 cm s^−1^.** (A) Whole-body and pectoral fin (left, red; right, blue) kinematics at 0.5 s intervals over a 2 s Kármán gaiting bout. (B) A zoomed in triplet of pectoral fin activity over a 0.2 s interval in which the left pectoral fin is abducted in image 1 to image 2 and then adducted in image 3. (C) Muscle activity of the left pectoral fin during this Kármán gaiting sequence; the shaded area corresponds to the triplet (B) and shows that the abductor muscles are initially used to extend the fin, then the adductors actively tuck the fin. (D) Muscle activity from the right pectoral fin shows no activity throughout the entire sequence.

**Fig. 8.**
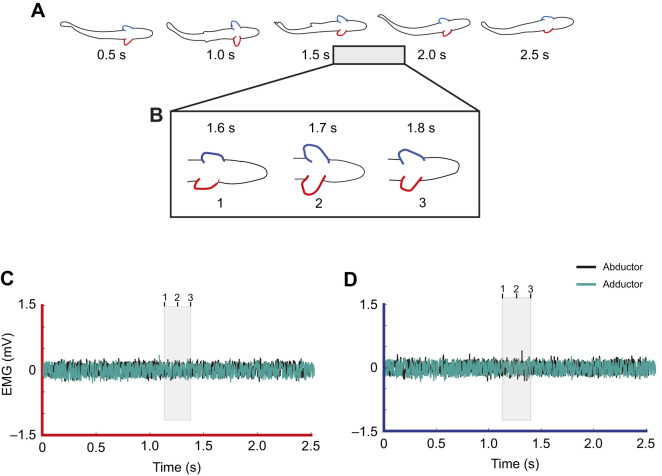
**Pectoral fin and whole-body kinematics in the absence of pectoral fin muscle activity for a trout Kármán gaiting over a 2 s interval at a flow speed of 65 cm s^−1^**. Data are for the same individual as in Fig. 7. (A) Whole-body and pectoral fin (left, red; right, blue) kinematics at 0.5 s intervals over a 2 s Kármán gaiting bout. (B) A zoomed in triplet of pectoral fin activity over a 0.2 s interval in which both the left and right pectoral fins are fully abducted (image 1 to image 2) and then are slightly adducted (image 3). (C) Muscle activity of the left pectoral fin during this Kármán gaiting sequence; the shaded area corresponds to the triplet (B) and shows no activity from either the abductor muscles or adductor muscles as the fin is fully abducted then slightly adducted. Furthermore, no muscle activity was observed throughout the entire Kármán gaiting sequence (A) for the left pectoral fin. (D) No muscle activity was observed throughout the entire Kármán gaiting sequence (A) for the right pectoral fin.

The pattern of muscle activity for fin extensions during Kármán gaiting was more nuanced than that for braking behaviors. In 80% of all fin extensions during Kármán gaiting, at least one pectoral fin muscle group (abductor or adductor) showed activity on either side of the body within a 0.2 s window prior to maximal fin extension (Table 1, onset). In the 0.2 s window after maximal fin extension, 83% of all fin extension events showed activity from at least one muscle group on either side of the body (Table 1, offset). Interestingly, less than 50% of fin extension events had any muscle activity on the same side of the extending fin either prior to or after maximal extension (Tables 1 and 2). Furthermore, around 20% of the time, no EMG activity was observed on either side of the fish either prior to or after maximal fin extension (Tables 1 and 2, Figs 6 and 8). An example of this type of Kármán gaiting sequence is shown in Fig. 6, where the left pectoral fin is extended as the trout moves from right to left across the von Kármán vortex street. The EMG trace from the left pectoral fin (Fig. 6C) showed no activity from either the abductor or adductor musculature as this extension occurred. There was also no muscle activity observed in the right abductor and adductor musculature during this entire sequence (Fig. 6B). In other instances (Fig. 7), fin movement during Kármán gaiting followed a more conventional pattern of muscle activation. In this sequence, activity from the abductor musculature facilitates pectoral fin extension and subsequent activity from the adductor musculature tucks the fin. Interestingly, the same individual fish from Fig. 7 at the same flow speed (65 cm s^−1^; 3 *L* s^−1^) was also observed extending and holding out its pectoral fin in flow without any corresponding activity from the abductor or adductor musculature (Fig. 8). These examples highlight the complex and often times inconsistent patterns of muscle activation and movement of an appendage in turbulent flow regimes.

**Table 2.**
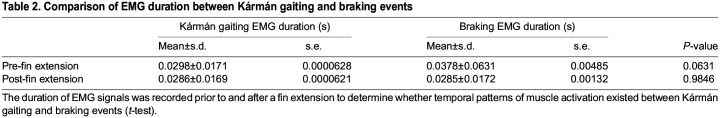
Comparison of EMG duration between Kármán gaiting and braking events

## DISCUSSION

Rainbow trout station holding in a von Kármán vortex street behind a cylinder used their pectoral fin during both braking and Kármán gaiting (Fig. 2), which take place in different regions around a cylinder in flow. During each of these behaviors, the pectoral fins showed distinct passive and active muscle activity patterns that mediated whole-body movements. Within each behavior, pectoral fins exhibited a large variation of movement patterns.

### Braking

Braking events were generally observed when trout were positioned 2–3 cylinder diameters (10–15 cm) downstream of the cylinder. Suction effects from fluid separation occur in the range of *Re* values seen in our experiments (*Re*=2.5×10^4^ to 4.8×10^4^) (Zdravkovich, 1997; Liao et al., 2003a).

Trout used pectoral fin activity to rapidly decelerate and control their downstream positioning behind a cylinder in flow. Braking allowed fish to stop their forward momentum to avoid collision with the cylinder (Fig. 4A) and enabled favorable downstream positioning to facilitate holding station for prolonged time periods.

Braking consisted of two stages of pectoral fin movements: (1) fins were abducted along the fish's long axis, which elevated and protracted the trailing edges (Drucker and Lauder, 2003) and (2) fins were then held abducted against the flow until the fish's body began drifting downstream. When the body was located in the centerline of the cylinder, braking consisted of the simultaneous extension of both fins about 70% of the time. Braking using a single fin occurred about 30% of the time when the body was off-axis from the cylinder's centerline. Single fin abduction was correlated to the cessation of the trout's forward movement when it approached one of the lateral edges of the cylinder.

Proper downstream positioning in a von Kármán vortex street is critical to prolonged station holding. Within the cylinder's vortex street, there exists a ‘saddle point’ region with optimal conditions that allows the flexible fish body to harness the energy of vortices for passive forward propulsion (Liao et al., 2003b; Beal et al., 2006). We hypothesize that the pectoral fins are primary drag-producing control surfaces that fish use to position themselves appropriately in the vortex street to maintain Kármán gaiting. While we observed a strong correlation between fin abduction and changes in whole-body velocity, we cannot rule out the contribution of the body and other fins in drag production.

Braking movements required active recruitment from both the abductor and adductor musculature. Abductor muscles were active throughout stage 1 of braking, during which the fins were rapidly extended (Fig. 5). The adductor musculature was primarily active during stage 2, when the extended fin was maintained out against the flow. We hypothesize that this activity from the adductor musculature aids in holding the fin out in flow, either by isometrically resisting abductor activity to hold the fin in place or by resisting the suction effect of low-pressure regions near the cylinder. It is also possible that prolonged adductor activity during braking could serve to modify the three-dimensional shape of the pectoral fin. This is consistent with results from trout pelvic fins that reveal that the adductor musculature can pull on the fin to fan out the outermost rays (Standen, 2010).

While the pattern of adductor activity during braking deviates from previous pectoral fin work, where adductor activity initiates the movement of the fin back towards the body axis (Westneat, 1996), studies in birds reveal that wing muscles can serve multiple functions during different phases of locomotion (Dial, 1992). Muscle function is dependent upon environmental conditions because external forces can strongly influence patterns of muscle activity (Liao et al., 2003b; Beal et al., 2006; Biewener and Daley, 2007; Li et al., 2013). This is especially relevant in the presence of external fluid forces, such as experienced in moving fluids, where it is possible that muscles can lengthen during contraction (Hill, 1938; Huxley, 1957). Because the relative movement of the pectoral fin can depend on the environmental fluid forces it experiences, it is difficult to determine whether the fin's position during braking is actively maintained by the adductor musculature or is the result of fluid forces resisting adduction.

Fins take on new capabilities in current that do not exist in still water (Webb, 1984; Westneat et al., 2004; Drucker et al., 2005). A fin held out from the body becomes a thrust- or drag-producing surface in the presence of flow (Webb, 1984, 2002; Blake, 2004). The significance of these forces for fish in flow is well established. Fins can be used as control surfaces to create trimming forces that interact with the passive forces generated by a fish's body shape and posture to help damp and self-correct yawing, heaving and pitching forces experienced in turbulent flows (Webb, 2002). Fish that live in turbulent environments such as chub and salmon rely on trimming forces to maintain dynamic stability and to enhance station-holding behaviors (Arnold et al., 1991; Webb, 1998). We show for the first time that the underlying muscle activity associated with trimming movements is complex and variable depending on local hydrodynamic conditions and cannot be predicted from previous pectoral fin activation studies (Westneat, 1996; Westneat et al., 2004). This complexity is likely the result of the nuanced interplay between three-dimensional forces created by cylinder vortices and the actuation and compliance of pectoral fin rays (Fish and Lauder, 2006).

### Kármán gaiting

Unlike braking, Kármán gaiting trout typically used their left and right pectoral fins asynchronously to hold position behind the cylinder (Fig. 3). When a fin was extended, it was correlated to the movement of the body towards that same side (Fig. 4). These fin movements appeared to be corrective, positioning the body towards the centerline of the cylinder wake. Pectoral fin movements varied widely during Kármán gaiting, with quick, single-fin extensions that immediately preceded pivoting adjustments, and prolonged, single-fin extensions mediating lateral movement across the entire vortex street (Figs 4B and 6).

**Fig. 9.**
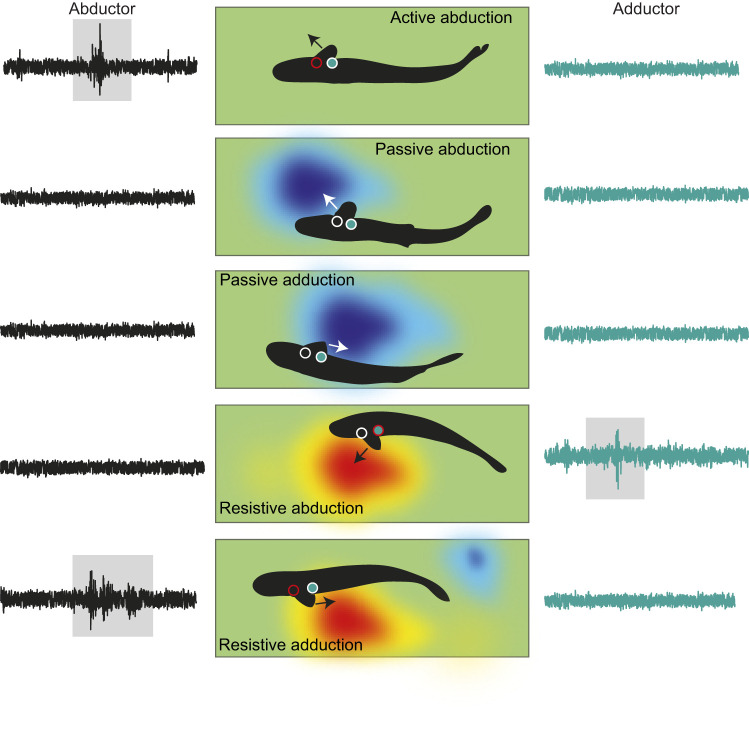
**Experimental kinematic and pectoral fin muscle recordings with hypothesized location of vortices in order to understand passive movement of the pectoral fins.** From top to bottom, active abduction relies on the abductor muscles to actively extend the pectoral fin. Passive abduction and adduction were also observed and are hypothesized to arise from the low pressure of vortices, which because of their strength can induce passive fin movement. In some cases, antagonistic muscle groups were active during fin movement (resistive abduction and adduction). Both passive movement and antagonistic muscle activation may result in fin extension in flow. Shaded areas highlight muscle activity.

We observed a complex relationship between pectoral fin motion and muscle activation during Kármán gaiting. Some Kármán gaiting sequences revealed canonical pectoral fin activity whereby abductor activation extended fins away from the body and adductor activation retracted the fin against the body (Fig. 7). However, in other sequences, pectoral fin extensions away from or towards the body had little to no corresponding muscle activity (Figs 6 and 8). In these instances, we hypothesize that the fin is being moved passively by the low-pressure cylinder vortices (Fig. 9). At times, it appears that antagonistic muscle groups hold the fin steady in flow (Fig. 9).

While previous studies have elegantly demonstrated how pectoral fins move to generate vortices (Drucker and Lauder, 1999; Wilga and Lauder, 2000; Lauder and Drucker, 2004; Lauder and Tytell, 2005), no work has examined how pre-existing vortices in the environment affect pectoral fin movement. We have found that in a von Kármán vortex street, pectoral fin movement does not always correlate with muscle activation, unlike in stagnant water or uniform flows (Westneat, 1996; Westneat et al., 2004). We observed that, just as passive or actuated mechanisms in other animals are used to navigate unsteady environments (Dial, 1992; Full et al., 1998; Biewener, 2003; Biewener and Daley, 2007), pectoral fins can move with or without any muscle activity. Passive fin movements occur when the body is located in certain regions of the vortex street, where the fins are in close proximity to the shed cylinder vortices. The low-pressure vortices can then exert an external force onto the fin to influence its movement. In this way, the pectoral fins may at times be passively interacting with vortices in much the same way that the whole body has been described to interact with the vortices (Liao et al., 2003a,b; Liao, 2004). The pectoral fins could be considered as small foils acting on top of the larger, moving foil of the body. While this complex interaction is not yet understood, we provide evidence here that during Kármán gaiting the pectoral fins are the key control surfaces that position the body to be able to extract energy from cylinder vortices.

Substantial intra- and inter-individual variation in pectoral fin movements and muscle activity likely reflects complex fluid–fin scenarios (Figs 5, 7 and 8; Fig. S3). Unlike terrestrial locomotion, where gravity influences the muscle activity of paired appendages to generate whole-body movements and stabilize perturbations (Cavagna et al., 1977; Winters et al., 2000; Biewener and Daley, 2007), animals moving through fluids are suspended in their medium, providing greater degrees of freedom for appendage activity. Muscle contraction can be decoupled from appendage movement in ways that could exceed terrestrial systems, given that in fluids, small changes in surface area and angle of attack of a flexible appendage can quickly generate either lift or drag (Daniel, 1984; Wilga and Lauder, 2000; Blevins and Lauder, 2012). For example, trimming movements in paired fins arise from underlying muscle activity that holds the fin stationary and extended into flow, and therefore in a position to generate lift and drag (Webb, 2002). Like the locomotory function of pectoral fins in fishes, the wings of flying birds also help them stay aloft and moving forward in a fluid, albeit at a higher Reynolds number regime. Birds have been shown to maintain their feathers extended for flight through a skeletal linkage system, where the passive extension of the elbow joint extends the wrist and spreads the feathers (Headley, 1895; Dial, 1992). We suggest that across the diversity of fishes, as species ranging from sturgeons to sea robins and tunas specialize to maintain their fins extended (Gleiss et al., 2019), similar passive, skeletal mechanisms may be discovered that could augment the variation in muscle activity patterns we observed in this study.

The general properties of von Kármán vortex streets are well known (Zdravkovich, 1997; Liao et al., 2003a,b; Beal et al., 2006), allowing for the correlation of fin kinematics and underlying muscle activity with certain hydrodynamic features. Our approach provides insight into how paired fins interact with unsteady flows, but is limited in that we did not measure instantaneous interactions between pectoral fins and local hydrodynamic conditions. We encourage future studies to employ an approach using simultaneous kinematics, EMG and particle image velocimetry to further unravel how fins and muscles interact with complex fluid environments.

### Pectoral fins as sensors in von Kármán vortex streets

Investigations into the function of pectoral fins have concentrated on their role as propulsors and control surfaces (Webb, 1973; Geerlink, 1986; Drucker and Jensen, 1996; Walker and Westneat, 1997; Drucker and Lauder, 1999; Wilga and Lauder, 2000; Lauder and Drucker, 2004). However, pectoral fins also play an important role in sensing (Flammang and Lauder, 2013; Aiello et al., 2018; Hale, 2021). Individual fin rays of the pectoral fin are innervated by afferent sensory fibers that encode the amplitude and velocity of fin ray bending (Williams et al., 2013). This pattern of fin afferent innervation is consistent across bony fishes, suggesting that sensory feedback is a fundamental feature of paired fins (Aiello et al., 2018). For example, the pectoral fins in the round goby (*Neogobius melanostomus*), a bottom-dwelling species, have been shown to encode the surface texture of benthic substrates. Remarkably, these fin ray afferents can phase lock to the stimulus temporal frequency at a level that is comparable to the primate hand (Hardy and Hale, 2020; Hale, 2021). Even non-benthic fishes rely on sensory feedback from their pectoral fins to facilitate locomotion. When pectoral fin ray afferents in labriform swimming parrotfish (*Scarus quoyi*) are ablated, their three-dimensional movement and underlying muscle activity are substantially altered (Aiello et al., 2020).

In this study, braking trout maintained their pectoral fins extended against the flow for prolonged periods. While these extensions are correlated to the stopping of forward velocity, they may also be sampling the environment to detect changes in fluid velocity and pressure. Detecting these changes, which could soon develop into larger perturbations, could help fish anticipate future instabilities and allow for course correction. In particular, vortices developing in the suction zone immediately downstream of a cylinder could shed into the wake, buffeting and destabilizing the body (Liao et al., 2003a). We suggest that the sensory function of pectoral fins may be particularly important when station holding and navigating in turbulent flows. The sensory capabilities of fins in response to hydrodynamic stimuli have not been well studied in non-labriform swimmers, providing a fertile and relevant topic for future studies of swimming. Although the focus of this study was to analyze the function of pectoral fins in turbulent flows, there is likely a powerful interplay between the sensing abilities of fins with other well-known mechanosensory structures such as the lateral line in detecting unique hydrodynamic signatures (Liao, 2006; Vanwalleghem et al., 2020; Hale, 2021). Ultimately, the propulsive and sensory functions of fish are inextricably linked, necessitating an integrative approach for any comprehensive understanding of locomotion through complex environments.

## Supplementary Material

10.1242/jexbio.246275_sup1 Supplementary information

## ECR Spotlight

This article has an associated ECR Spotlight interview with Brendan J. Gibbs.
