# Supplementary material for: Kinematics and muscle activity of pectoral fins in rainbow trout (Oncorhynchus mykiss) station holding in turbulent flow
Source: J Exp Biol. 2024 Mar 12;227(5):jeb246275. doi: 10.1242/jeb.246275 (PMC10984278; doi:10.1242/jeb.246275)
Supplement: Supplementary information [file jexbio-227-246275-s1.pdf]

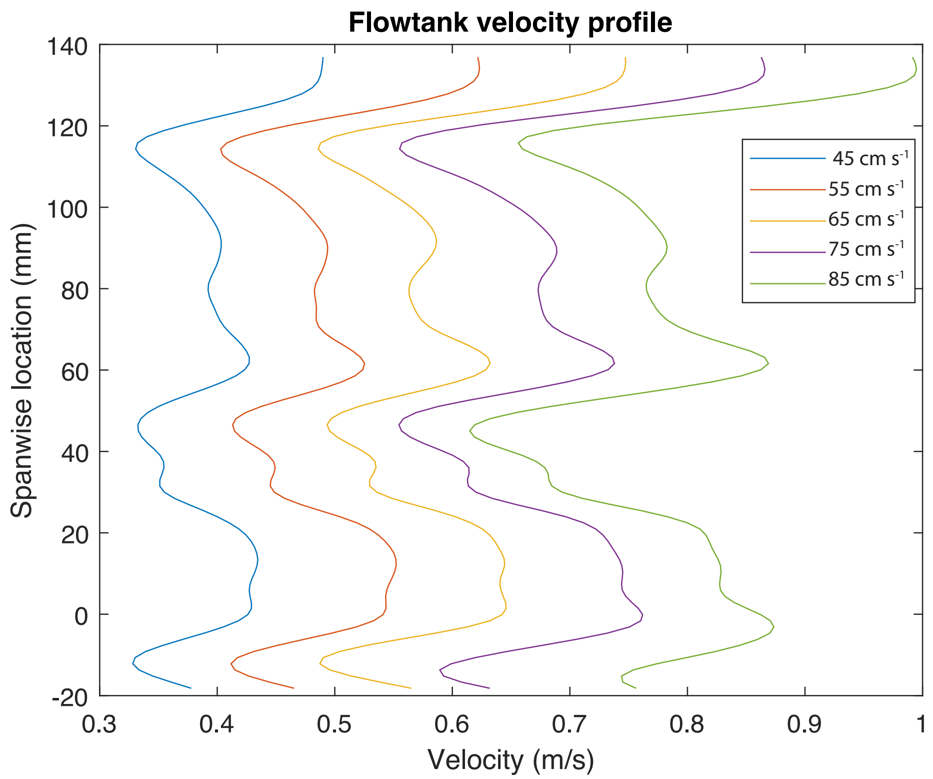

Fig. S1. Uniform velocity profile of the flow tank flume at our five tested flow speeds (45-85 cm s^{-1}) using PIV. The midline of the flow tank flume is at the spanwise location of 45 mm. It can be observed that there are lower recorded velocities at the spanwise locations of -10, 45 and 110 mm as these are the areas where are support baffles are located upstream.

Journal of Experimental Biology • Supplementary information

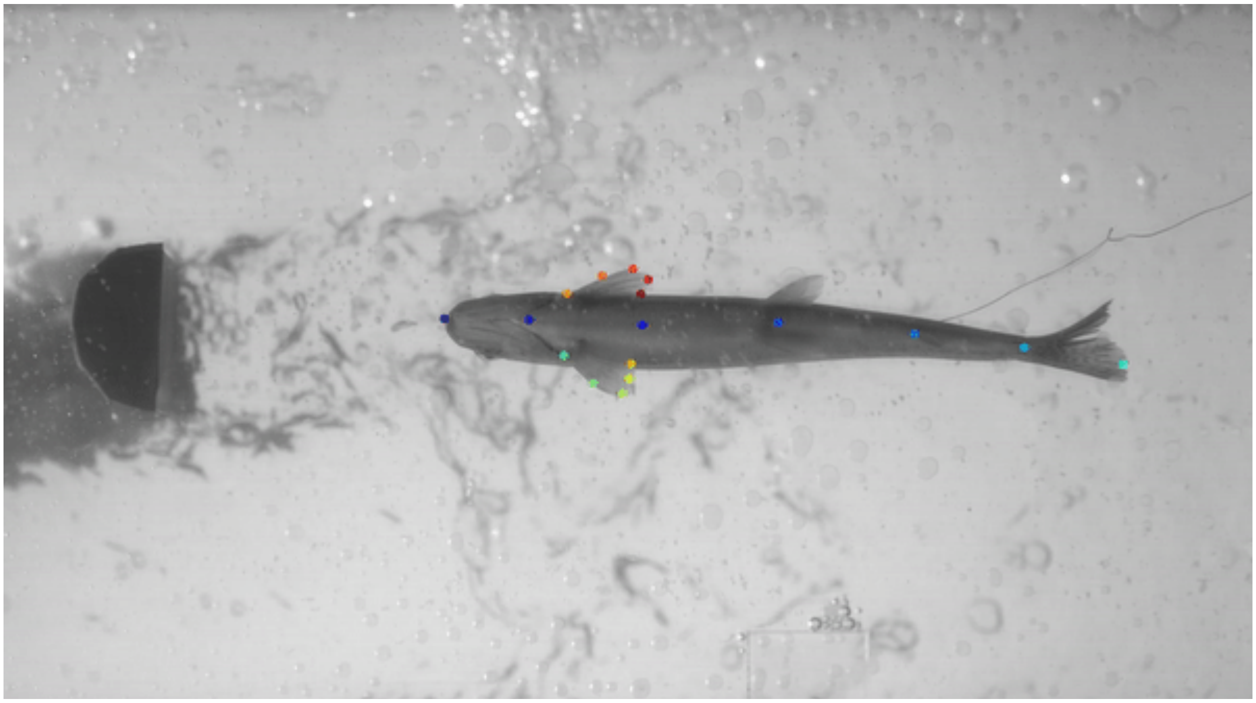

Fig. S2. Active tracking of fish kinematics using DeepLabCut. Five points on each pectoral fin (two at the fin base (points 1 and 5), one at the distalmost point of the leading edge (point 2), one at the distalmost point of the trailing edge (point 4), and one point at the longest radial (point 3)) were used to quantify fin movement as well as seven points on the midline (snout, gill arch, pectoral fin midpoint, pelvic fin girdle, anal fin, caudal fin base, caudal fin tip) to derive whole body kinematics

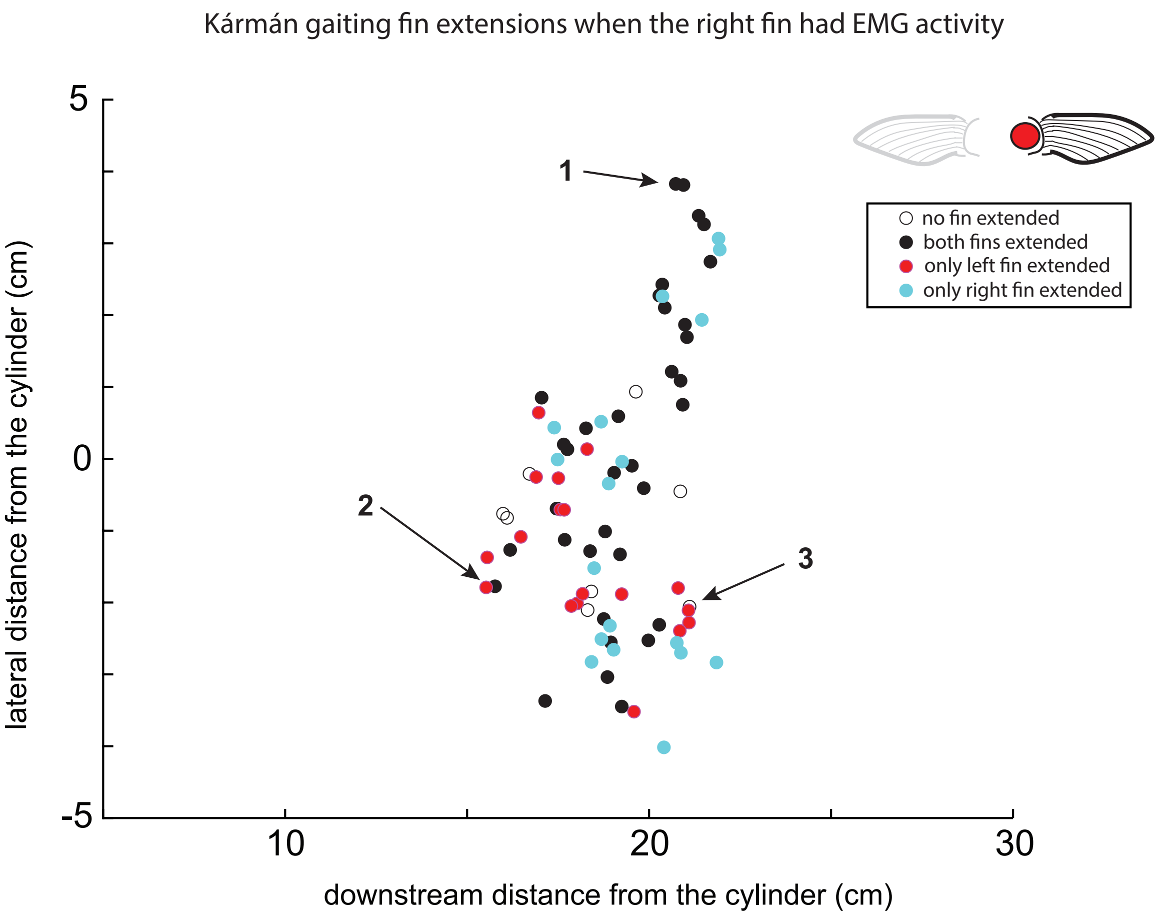

Fig. S3. Pectoral fin muscle activity, kinematics and center of mass relative to the cylinder (COM) during Kármán gaiting. All dots represent the trout's COM where EMG activity was observed from the right fin musculature. The different colored dots represent the kinematics of both fins during this muscle activity. At times, we observed a correlation between COM location and specific fin kinematics (Arrow 1). We also observed that fin kinematics could vary considerably when the COM was in the same location of a Kármán vortex street (Arrow 2; Arrow 3). These examples illustrate the spatial complexity of fin activation and movement in inherently unstable environments like a Kármán vortex street.

Journal of Experimental Biology • Supplementary information
